# API Development Increases Access to Shared Computing Resources at Boston University

**DOI:** 10.4236/jsea.2022.156011

**Published:** 2022-06-29

**Authors:** George Jones, Amanda E. Wakefield, Jeff Triplett, Kojo Idrissa, James Goebel, Dima Kozakov, Sandor Vajda

**Affiliations:** 1 Department of Applied Mathematics and Statistics, Stony Brook University, Stony Brook, USA; 2 Department of Chemistry, Boston University, Boston, USA; 3 Department of Biomedical Engineering, Boston University, Boston, USA; 4 Revolution Systems, LLC., Lawrance, USA; 5 College of Engineering, Boston University, Boston, USA

**Keywords:** API Framework, Open Source, High-Performance Computing, Software Architecture, Science and Engineering

## Abstract

Within the last few decades, increases in computational resources have contributed enormously to the progress of science and engineering (S & E). To continue making rapid advancements, the S & E community must be able to access computing resources. One way to provide such resources is through High-Performance Computing (HPC) centers. Many academic research institutions offer their own HPC Centers but struggle to make the computing resources easily accessible and user-friendly. Here we present SHABU, a RESTful Web API framework that enables S & E communities to access resources from Boston University’s Shared Computing Center (SCC). The SHABU requirements are derived from the use cases described in this work.

## 1. Introduction

Increases in computational resources have contributed enormously to the progress of science and engineering through the ability to generate, interpret, utilize, and share data quickly and cost-effectively. Over the last two decades, the development of High-Performance Computing (HPC) capabilities has been driven by the need for more powerful systems and applications. Significant improvements in technology have pushed the limits of HPC and have brought about large changes in scientific discovery. Specifically, it is now standard practice to include large-scale computational studies to assess if a theory is consistent with experimental results, question a large collection of data, or understand mechanisms through high precision simulations.

With the constant development of new algorithms and applications, it becomes imperative that users and applications can easily access computing resources, especially HPC resources. Many academic institutions, including Boston University (BU), provide HPC resources in the form of Shared Computing Centers (SCC) that enable students, staff, and faculty to run resource-intensive calculations vital for S & E. Increases in the types of users, including individuals and webservers, necessitate improved access to SCC resources. Before this work, access to the SCC at BU was limited to SSH/SCP protocols and required two-factor authentication of users. This created challenges for developing and maintaining S & E web servers that utilize the SCC computing resources.

Web Application Programming Interfaces (Web API) [1], a set of rules for how applications connect and communicate, provides developers with frameworks for building HTTP-based services accessible by software applications. Current Web API development tends towards the Representational State Transfer (REST) [2] [3] [4] [5] [6] architectural style, which provides a high level of flexibility. RESTful API is a software design pattern that specifies a uniform and predefined collection of stateless operations. RESTful Web APIs have become a building block of web-based software development due to their interoperability between applications and systems over the web.

This work describes the SHared API at Boston University (SHABU) framework for creating REST-ful web APIs for high-performance computing (HPC) centers. The API generated by the SHABU framework provides an interface through which web servers can access HPC resources on the SCC. We set out to create a framework to meet the growing demands without causing delays for servers relying on the BU SCC for computing, interrupting normal user activities, or compromising security. Scientists and engineers require broadly accessible computational resources for effective work and collaborations. Therefore, computing system must be able to accommodate various inputs and perform necessary calculations. We have developed a customizable framework that can be deployed at HPC centers to enable access to various backend resources and services through a common web API. This effort aims to create an easily extendable service that can be plugged into multiple backend resources.

## 2. Design and Development

The recent addition of several servers using SCC resources combined with increases in the usage of existing servers has led to some problems. Historically, communications between servers and the SCC, including submissions, file transfers, and monitoring, were handled with SSH/SCP protocols. Increases in the number and usage of servers have led to substantial growth in the number of queries submitted to the SCC, which has created slowdown issues and connection issues. As a result, jobs consistently fail due to timeouts and longer than normal run times. In addition, recently improved security protocols for SCC users, including the introduction of two-factor authentication, hamper the functions of the servers. Currently, this is worked around by reducing security measures from specific IP addresses; however, this undermines the security efforts. To comply with the new regulations and ensure proper server functioning, we decided to introduce an API for submission, management, and monitoring of computing jobs from servers utilizing SCC resources.

We decided that an API would be the best option for enhancing access to computing resources on the SCC by servers at BU. To start this project, we searched existing open-source projects and code to find an API compatible with the software and architecture of the SCC. Despite the availability of several resource-sharing platforms [7] [8] [9] [10], there are no out-of-the-box solutions that meet the needs of the servers reliant upon the SCC. Therefore, we designed a framework, SHABU, for a centralized method for communicating with the SCC, which many servers can use hosted from any number of locations. SHABU must meet the following requirements:

1. Receive a job workflow and submit it to the queue, monitor the status until completion, and return the results to the server.
2. Easily incorporate additional servers and job workflows.
3. Handle multi-part workflows.
4. Allow for testing and development.
5. Maintain the security of the SCC.

Django, a Python-based web framework, was selected because it supports all required functionalities [11]. The connection between the API and the SCC was developed as a Docker volume to provide seamless security and access to resources [12]. Celery was used as an asynchronous job handler because it works well with Django in a Docker environment, and it can accommodate variability in the size and number of jobs [13].

## 3. Architecture

**Figure 1.**
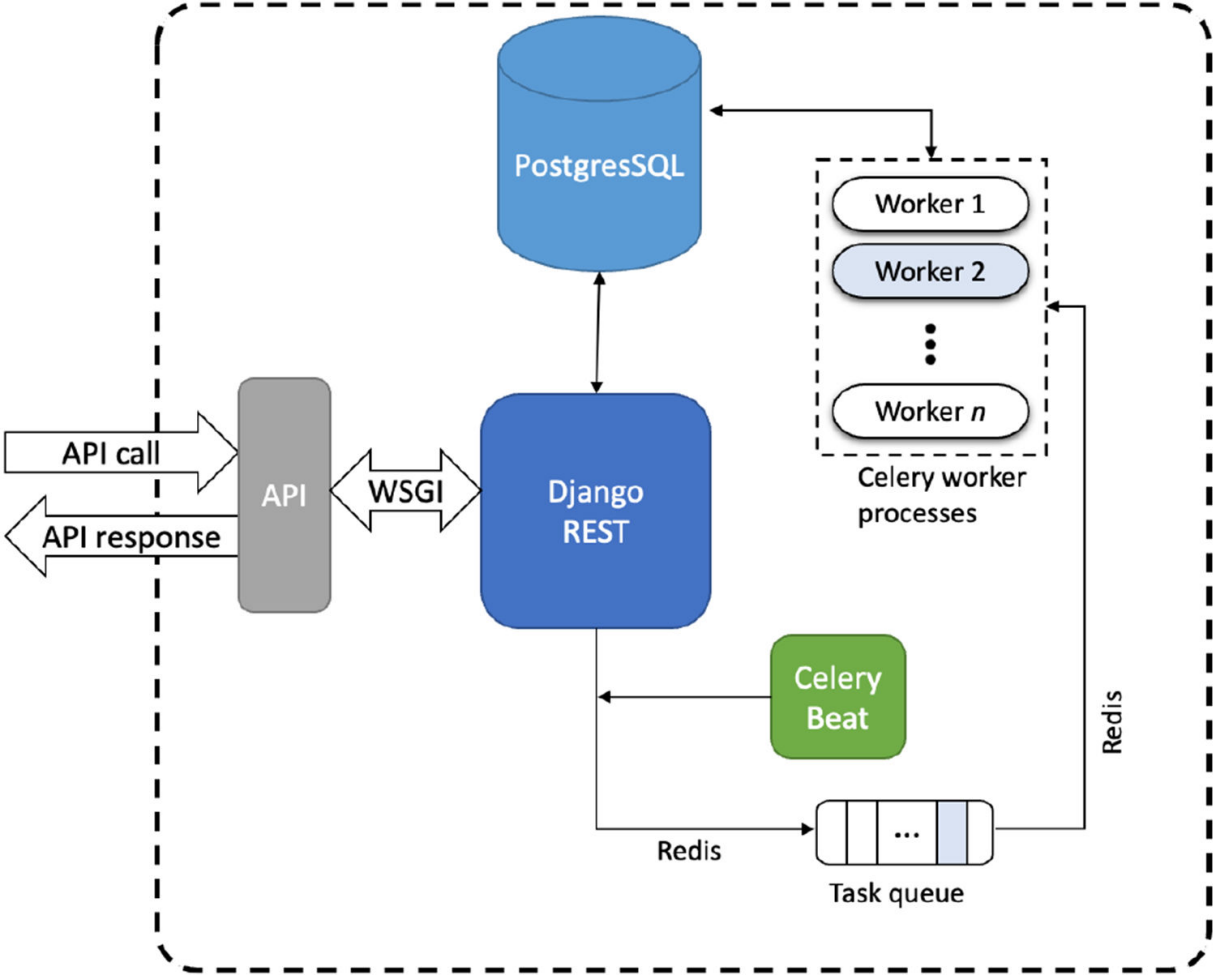
Information flow generated by the user.

SHABU provides users with web-based API endpoints, shown in Table 1, to access resources on the SCC. To achieve this, SHABU converts HTTP requests into workflows on the SCC. In the process of doing so, it requires data movement, user authentication, job management, and additional operations. The job object is core to SHABU’s functioning, and most of the architecture revolves around the management of proper resources, authentication, and handling of the jobs submitted. The job management system is outlined in Figure 1. SHABU is built using multiple open-source tools such as Django, Redis, Celery, Caddy, and Postgres [14] [15] [16] [17] [18]. The following subsections will present the SHABU/SCC connection, identity access management, API, job management, maintenance, and job execution.

### 3.1. SHABU/SCC Connection

SHABU accesses the SCC through an NFS Docker volume mounted inside the server’s Docker container. To facilitate communication between the GPFS file system, which SCC uses, and the NFS docker volume, the working directory was first made NFS accessible. The volume contained the SCC user authentication and was designed to provide a stable connection to SCC resources.

### 3.2. Identity Access Management

Identity access management (IAM) protocols have been set up to ensure the proper users have access to running commands on SCC. SHABU is designed to be an interface used for open access servers. The user setup and API restrictions put into place are designed to allow designated servers access. Restrictions fall into two main categories: user-based and SCC-based.

User restrictions are based on user accounts created on the SHABU site. Anyone on the SHABU site can create an account; however, to submit jobs to the API, a request must be made to add the user into an access group. Entry into the access group will allow the user to create an access token. These tokens are created using the rest_framework.authtoken module for Django. Once a token is created for a user, they can register an IP address where the server will be located. The IP address and token combination will allow users to access the server from the registered IP address.

SCC-based restrictions are based on SCC user accessibility. SHABU runs all SCC-based code through a single user with limited access. If a specific workflow requires libraries or executables to be made available, the user can contact the administrator. Environments can be created which cater to specific workflows.

### 3.3. API

SHABU provides a secure way to interface with job management services via an API. The API is hosted in a web-facing Docker container. Interactions with the API are verified using tokens and IP information. Once this verification process is complete, the request information is processed to ensure valid requests. A verified request is then passed to the corresponding service. The API is built using Django REST Framework. Swagger provides documentation for the API.

### 3.4. Job Management

**Figure 2.**
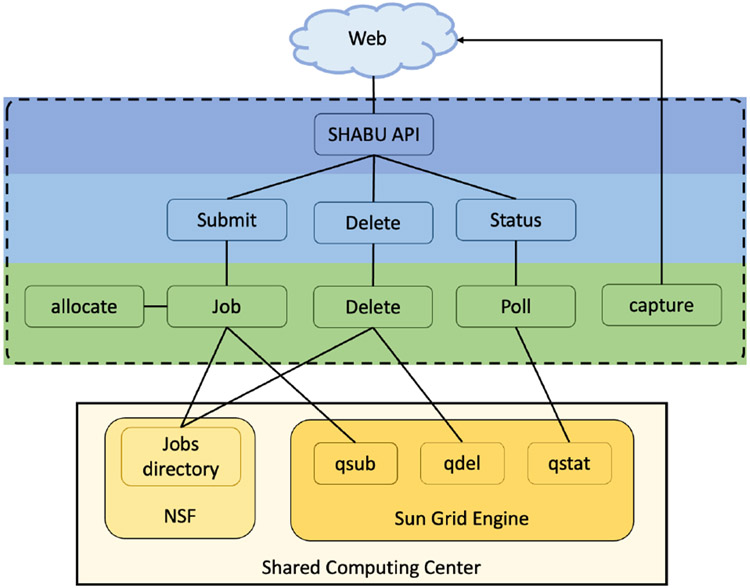
Workflow of user interactions. All users interact with the API (blue) to run commands based on API input (light blue). These commands generate tasks to run using celery (green) which interacts with the SCC (yellow), specifically the jobs directory and the SGE.

SHABU’s job management interactions, as outlined in Figure 2, include job submission, deletion, status check, and modification.

#### 3.4.1. Job Submission

When a user submits a job request to the API, the user is verified via their token and IP address. Verified requests generate an asynchronous task that completes the processing of the request. This task is submitted to the Celery worker queue and subsequently executed using Celery workers. The task creates a unique directory on the SCC using the NFS volume mount and unpacks the request methodology and supporting files into this directory. The request methodology is submitted to the SCC SGE queue to be run. The asynchronous task captures the SGE associated job id number and records it in the database.

#### 3.4.2. Job Deletion

When a user deletes a job, the API request is verified via the token and IP address of the user. Verified requests result in an asynchronous task submitted to the Celery queue. The deletion task removes the job folder on the SCC and removes any jobs from the SGE queue. The status of the job will also be modified to “Deleted.”

#### 3.4.3. Job Status

The request is first verified when a user sends a job status query to the API. Verified requests return a JSON package that contains details of the job. These details include the status of the job on the SHABU queue, the job status on the SGE queue, and the SGE id.

#### 3.4.4. Job Modification

When a user sends a job modification query to the API, it is first verified. A request will include the job SHABU id and modifications to the job parameters. Once the request is verified, the job’s details will be updated using the supplied information.

### 3.5. Maintenance

The job submission task is complete once the job is submitted to the SGE queue. The task of updating jobs relies on periodic tasks, which can be classified under maintenance. The maintenance tasks are run using Celery Beats.

#### 3.5.1. Allocating Jobs

This task queries the database to see if there are any jobs in the SHABU queue and how many jobs are active. If there are jobs in the queue and the number of jobs active is less than the set maximum number of jobs, this task will activate jobs in the queue. This activation starts the asynchronous task, which runs the job methodology outlined in job management.

#### 3.5.2. Poll Job

This task periodically queries the SGE queue to get the status of jobs running on the SCC. The SGE queue is queried using qstat for user-specific jobs. The task iterates through the jobs in the SHABU queue; if the sge_id is in the SGE query results, the SGE status of the job is updated. If a job is no longer found in the SGE queue, the status in the SHABU queue is updated to complete.

#### 3.5.3. Capture Job Output

This task periodically checks the jobs on SHABU to see if jobs have been completed or failed. This task creates an output file package using tar for jobs that meet this criterion. Each of the webserver API users has a webhook address that is used to send the output files to the corresponding server. This task will create an output tar file; once the output file is created, the working files on the SCC are deleted. A webhook is then sent to the specified address to send the output files to the server.

#### 3.5.4. Cleaning

This task will remove jobs that are older than the specified retention date. This task sets the database status to DELETED for each expired job and removes all job-related files from the SCC NFS.

## 4. Deployment

The final step in software development is deployment. Effortless and accurate deployment is imperative for the usefulness of the software. Deployment involves provisioning the production environment with the required operating system, packages, libraries, and configuration files and brings all these components together to work as one unified system.

We have chosen to deploy SHABU with Docker. Docker enables the packaging of required dependencies, including configuration files and libraries in clean, redistributable Docker containers. The execution of these containers reproduces the exact production environment on a user’s machine. We provide four separate docker containers for the RESTful API, Redis, Celery, and Celery Beats. This allows us to isolate the components and choose the appropriate software stack for each component.

**Figure 3.**
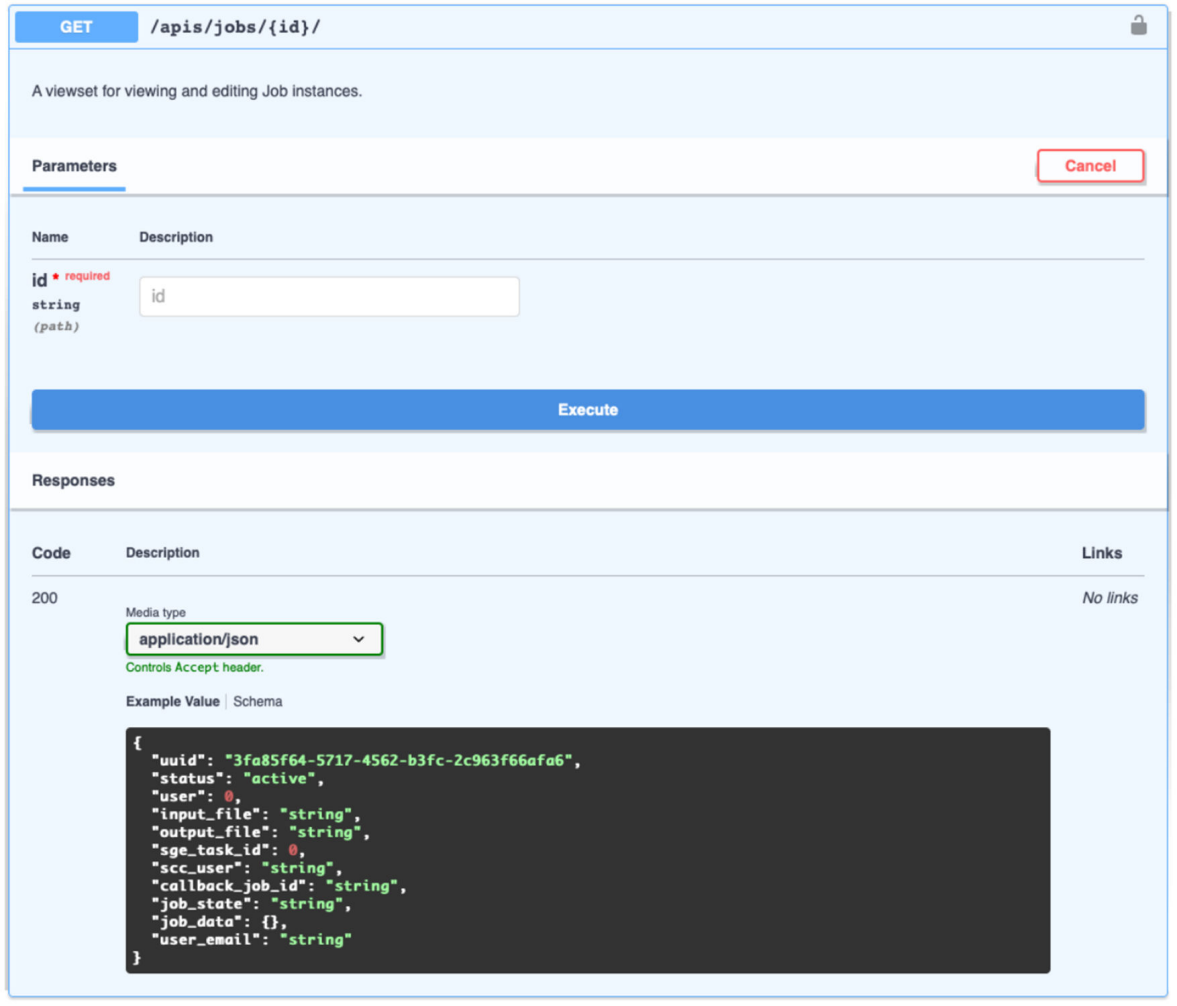
Looking up a job with the swagger UI documentation for the “/apis/jobs/<id>/” endpoint. The Swagger UI provides a webpage for users to explore the API interactively.

**Figure 4.**
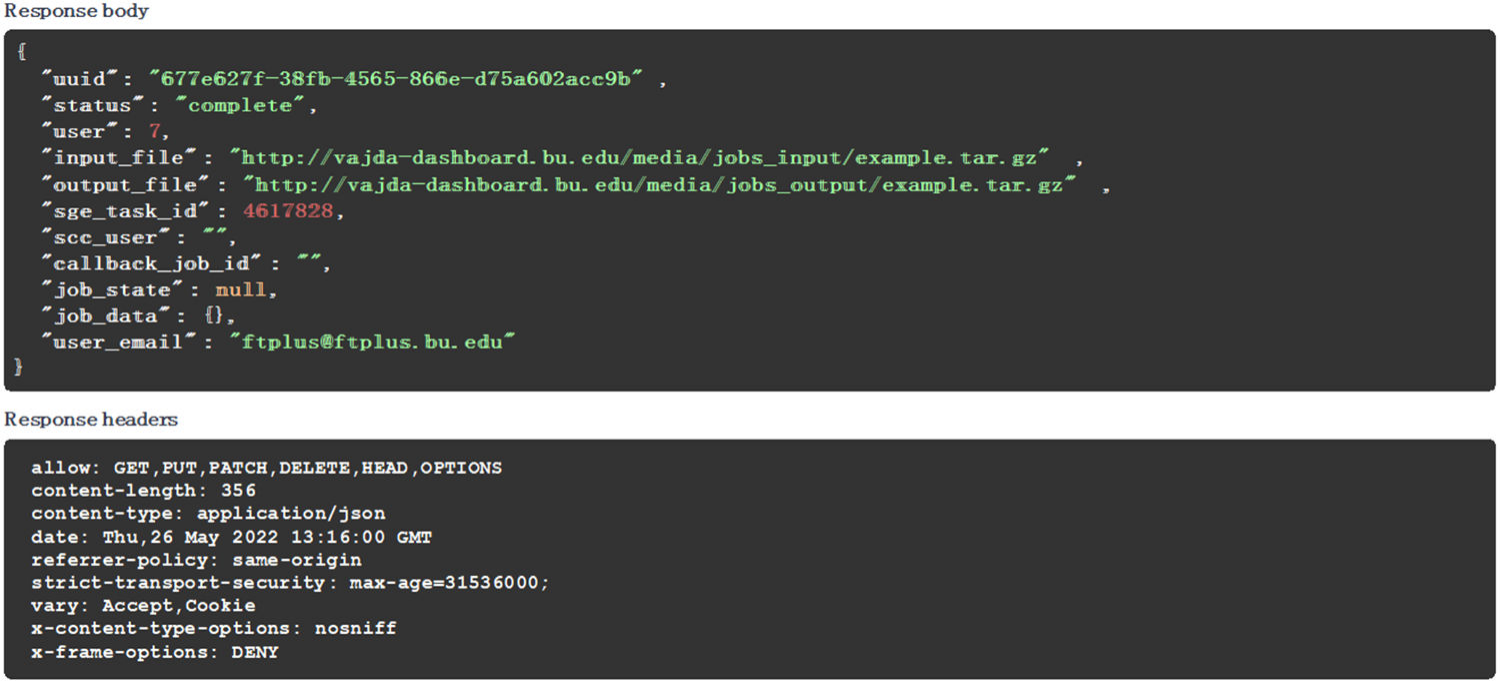
Result of looking up a job using the swagger UI. The results were obtained after querying the “/apis/jobs/<id>/” API endpoint. The response body section shows the JSON response received from the API, and the response headers section shows the HTTP headers from the received request.

The API documentation is provided via the Swagger API documentation tool. The Swagger user interface (UI) allows users to explore the API and run test queries. For example, as seen in Figure 3, the UI can be used to look up a job by its id. Figure 4 shows the JSON response code and headers returned by the server.

## 5. Use Cases

### 5.1. Predicting Protein-Protein Binding Poses

ClusPro is a web server that uses rigid-body docking to find energetically favorable poses for submitted proteins [19]. Protein-protein interactions (PPI) allow for the basic functioning of cells, and they are also essential in larger biological systems. X-ray crystallography is the gold standard for understanding and confirming PPIs; however, the method is complicated and time-consuming [20]. Protein docking is a computational tool that provides a low-cost method of generating potential poses for PPIs that can be validated experimentally [21]. ClusPro provides a means to dock submitted proteins.

The main utility of ClusPro is to dock two user-defined protein structures. The main workflow involves taking in the user-defined structures, preparing and docking the structures, and generating the results for the user. These steps can be modified and must be flexible to fit the desired needs. To provide flexibility, ClusPro creates a workflow based on user input. This workflow was previously run using SSH/SCP protocols to transfer files to and from the SCC and check on the status of the job. Before SHABU, each job required periodic queries to the SCC to check the status. This system did not scale well as the jobs were monitored on an individual basis and became more problematic as the number of submitted jobs continued to increase. This system led to slowdowns on both the ClusPro server and the SCC. Switching the ClusPro server from using the SSH/SCP protocols to using the API provided by the SHABU framework has drastically reduced the slowdowns on the server and the SCC.

ClusPro packages a workflow and the necessary support files and sends the file via a POST request to the API. The API receives the package and submits the workflow to the SCC queue. ClusPro can query SHABU to inform the user of the status of the job; however, SHABU asynchronous tasks monitor the status of all jobs and update the ClusPro database when there are changes to the status. Once a job has been completed, the resulting files are compressed and sent to the ClusPro server to be made available to the ClusPro user.

### 5.2. Identifying Hot Spots on Proteins

Protein-small molecule interactions are central to biological processes; therefore, understanding these interactions is an important research topic [22]. It is well established that regions of proteins that are capable of binding multiple, fragment-sized molecules, often referred to as hot spots, are the regions that contribute most significantly to protein-ligand binding energetics. Therefore, detection of binding sites on proteins allows for insight into which interactions contribute the most favorably to binding [23]. Computational hot spot detection methods such as FTMove, identify protein hot spots via the docking of molecular fragments to the protein [24].

FTMove is a web server that identifies protein hot spots by utilizing structural information gained from homology models of a submitted structure [25]. This allows for identifying dynamic sites, such as allosteric or cryptic, that can be overlooked if only a single structure is analyzed. Prior to accessing the SCC resources via an API, FTMove jobs were run by submitting individual jobs to the SCC for each docking process, followed by post-processing on the FTMove server; job monitoring was also done individually. The individual submission and monitoring of jobs are problematic. Besides the problems previously mentioned with the ClusPro server, FTMove has to transfer significantly more files to and from the SCC. Post-processing is therefore completed locally on the FTMove server. However, using the API allows for an array job to be submitted which runs all the docking jobs, compiles the results, and returns a single results file regardless of the number of homology models provided by the user. This helps keep the FTMove server independent of the FTMove algorithm, as is best practice.

## 6. Conclusions and Future Work

In this work, we present SHABU, a RESTful Web API framework that allows access to High-Performance Computing resources and services available from the Shared Computing Center at Boston University. We intend to use SHABU with the use cases presented in this paper. As new use cases emerge, new requirements will be requested for SHABU. There are plans to expand the framework to work across many High-Performance computing platforms, including Stony Brook University’s SeaWulf center and cloud-based services such as Amazon Web Services (AWS).

## Figures and Tables

**Table 1.** Endpoints provided by the API.

Endpoints	Description/Summary
*/apis/users*	Retrieve a list of all the users
*/apis/jobs*	Add a new Job instance to the task queue
*/apis/jobs/<job_id>*	Update or Delete a Job instance
*/apis/jobs/stats/*	Get a list of all the jobs and their current statuses
